# Supplementary material for: Differences in the growth rate and immune strategies of farmed and wild mallard populations
Source: PLoS One. 2020 Aug 31;15(8):e0236583. doi: 10.1371/journal.pone.0236583 (PMC7458304; doi:10.1371/journal.pone.0236583)
Supplement: Attachment — Submitted filename: ResponseToReviewers.docx [file pone.0236583.s005.docx]

Magdalena Ruiz-Rodriguez
Academic Editor
PLOS ONE

Dear Professor Ruiz-Rodriguez,

herein, we are re-submitting our manuscript **PONE-D-20-03121** “Differences in the growth rate and immune strategies of farmed and wild mallard populations” to be further considered for publication in the *PLOS ONE*. We have conducted changes in the manuscript according to your comments and suggestions of the Reviewer 2. Detailed list of our responses and text changes are provided bellow.

On behalf of all authors,

Yours sincerely,

Jana Svobodová (corresponding author)

Jana Svobodová

Department of Ecology

Czech University of Life Sciences Prague

# Kamýcká 129

# CZ- 165 00 Prague

## Czech Republic, Europe

Phone: +420 224 383 852

svobodovajana@fzp.czu.cz

**Responses to Editor's Comments**

This new version has been really improved with the changes performed according to reviewers suggestions.

In addition to the comments to this new version made by the Reviewer 2, I would like to add the following:

L36: change “demonstrated” to “demonstrate”

**Response:** It has been corrected (l. 36).

L373: change “worlds” to “words”

**Response:** It has been corrected (l. 373).

L420: change compare to “compared”

**Response:** It has been corrected (l. 420).


**Responses to Reviewer**'**s comments**

Reviewer #2: I am satisfied with the answers to my questions and comments on the first draft of the manuscript. I feel like the authors have listened to the comments and improved the manuscript. I still have some minor suggestions for the authors to deal with.

Abstract
L34: Change “parameters and a higher growth rates and higher complement activity” to “parameters, a higher growth rate, and higher complement activity”.

**Response:** It has been changed (l. 34).

L37: Change “population” to “populations”

**Response:** It has been corrected (l. 37).

L38: Change “prose” to “argue”.

**Response:** It has been changed (l. 38).

L39: Add “the” before “breeding population”.

**Response:** It has been added (l. 39).

Introduction
L41: delete “as” before “hand-reared”.

**Response:** It has been deleted (l. 41).

L59: add “of” before “the most widespread”

**Response:** It has been added (l. 59).

Material & methods
L118: Maybe there was some misunderstanding. I still think that you need to give the number of collected eggs here in the m&m. If you will present that not all eggs hatched successfully, that probably belongs in the results. The most important thing is that the numbers add up.

**Response:** Numbers of collected eggs represented wild and farmed populations have been added in the text (l. 118 and 124).

L157: Check spelling of “leukocyte” in whole document, be consistent.

**Response:** It has been corrected (l. 157). “Leukocytes” is consistently used through the manuscript.

L208: Change “mm3” to “mm3”.

**Response:** It has been changed to “mm^3^” (l. 208-209).

Results
L249-253: This sentence about the slope of body mass and tarsus length is not connected to the t-test in the following sentence, right? The t-test is just for the final measurements of body mass and tarsus? Maybe start with that the final measurements were significantly different and give the t-test and then continue with that also the slopes were different. Or, give test-values for the first statement that the slopes were different.
Also suggested change of sentence, from ”the average body mass was 321.6 ± 7.6 g (mean ± S.E) and tarsus length 46.4 ± 0.3 mm in 20-day-old farmed duckling, compared with 238.9 ± 11.3 g and 42.2 ± 0.5 mm (t-test: p < 0.0001 in both cases), respectively, in wild ducklings of the same age” to: ”body mass and tarsus length differed significantly between farmed and wild 20-day-old ducklings (mean ± S.E: 321.6 ± 7.6 g, 46.4 ± 0.3 mm, and 238.9 ± 11.3 g, 42.2 ± 0.5 mm, respectively; t-test: p < 0.0001 in both cases).”

**Response:** The sentence has been changed according to suggestion of the reviewer (l. 251-254).

L300-306: Be consistent when writing their age (3-day-old, 9-days old, 15-day- old, 3-days-old etc). Check whole manuscript.

**Response:** It has been changed to “3-day-old”, “9-day-old” and “15-day-old” respectively (l. 302-307) throughout the manuscript.

Discussion
L354: Change “non-sceletal” to “non-skeletal”.

**Response:** It has been changed (l. 355).

L359: Change “means” to “mean”.

**Response:** It has been corrected (l. 360).

L360: Change “with” to “by”.

**Response:** It has been changed (l. 361).
